# 1351. Predictors of Loss of Infectivity Among Healthcare Workers with Primary and Recurrent *SARS-CoV-2* infection: An Observational Cohort Study

**DOI:** 10.1093/ofid/ofad500.1188

**Published:** 2023-11-27

**Authors:** Stefka Dzieciolowska, Yves Longtin, Hugues Charest, Tonya Roy, Judith Fafard, Ines Levade, Jean Longtin, Leighanne Parkes, Jasmin Villeneuve, Patrice Savard, Jacques Corbeil, Gaston De Serres

**Affiliations:** McGill University Faculty of Medicine, Montreal, Quebec, Canada; Jewish General Hospital, Montreal, Montreal, QC, Canada; Laboratoire de Santé Publique du Québec, Institut National de Santé Publique du Québec, Montreal, Quebec, Canada; Laboratoire de Santé Publique du Québec, Institut National de Santé Publique du Québec, Montreal, Quebec, Canada; Laboratoire de Santé Publique du Québec, Institut National de Santé Publique du Québec, Montreal, Quebec, Canada; Laboratoire de Santé Publique du Québec, Institut National de Santé Publique du Québec, Montreal, Quebec, Canada; Centre Hospitalier Universitaire de Québec, Quebec City, Quebec, Canada; SMBD Jewish General Hospital, Montreal, Quebec, Canada; Institut National de Santé Publique du Québec, Quebec City, Quebec, Canada; Centre Hospitalier de l’Université de Montréal, Montreal, Quebec, Canada; Université Laval, Quebec City, Quebec, Canada; Institut National de Santé Publique du Québec, Université Laval, Quebec City, Quebec, Canada

## Abstract

**Background:**

Factors associated with loss of infectivity in healthcare workers (HCWs) with COVID-19 are poorly understood. Understanding predictive factors could help optimize return-to-work criteria and minimize absenteeism.

**Methods:**

Prospective observational cohort study of HCWs with COVID-19 conducted between Feb 20 2022 and March 6 2023 in 20 institutions in Montreal, Canada, with clinical/laboratory follow-up on Day 5, 7 and 10 of infection. Infectivity was determined by viral culture (Vero E6 cells) on nasopharyngeal swabs. Predictors of loss of infectivity were investigated by univariate and multivariate logistic regression.

**Results:**

Overall, 121 participants (79.3% female, mean age 40 years) were recruited. Most (n=107, 88.4%) had received ≥3 vaccines and 20 (16.5%) had a history of prior COVID-19. The proportion of HCWs with a positive viral culture decreased from 71.9% on day 5 of infection to 18.2% on day 10. The proportion of HCWs with a positive RT-PCR decreased from 93.3% (112/120) on day 5 (median Ct value, 23.4 [IQR, 20.6-27.9]) to 61.2% (74/120) on day 10 (median Ct value, 32.5 [IQR, 28.5 to undetectable]). Rapid antigen detection test (RADT) positivity decreased from 81.5% on day 5 to 34.2% on day 10.

Participants with recurrent COVID-19 had lower likelihood of infectivity at each visit (OR on day 5, 0.14; 95% CI 0.05-0.40; p< 0.001; OR on day 7, 0.04; 95% CI, 0.01-0.33; p=0.003) and none were infective on day 10 (p=0.02). At each visit, recurrent cases had higher median RT-PCR Ct values than primary infections (p< 0.03) and were more likely to have a negative RADT result (p< 0.01).

By multivariate analysis, ongoing infectivity was associated with a RT-PCR Ct value < 23 (adjusted OR [aOR] on day 5, 22.75; p< 0.001; aOR on Day 7, 182.30; p< 0.001; and aOR on Day 10; 24.71; p=0.02). A history of previous COVID-19 was associated with a lower probability of infectivity on Day 5 (aOR, 0.005; p=0.003). By contrast, symptom improvement (including fever) and RADT result were not independent predictors of loss of infectivity.

**Conclusion:**

A lower RT-PCR Ct value is associated with ongoing infectivity, whereas COVID-19 reinfection is a predictor of loss of infectivity. These findings could help optimize return-to-work algorithms.

**Disclosures:**

**All Authors**: No reported disclosures

